# Computer Vision and Robotics for Cultural Heritage: Theory and Applications

**DOI:** 10.3390/jimaging9010009

**Published:** 2022-12-30

**Authors:** Guillaume Caron, Olga Regina Pereira Bellon, Ilan Shimshoni

**Affiliations:** 1 MIS Laboratory, Université de Picardie Jules Verne, 80039 Amiens, France; 2 CNRS-AIST JRL (Joint Robotics Laboratory), IRL (International Research Laboratory), Tsukuba 305-8577, Japan; 3 Imago Research Group, Universidade Federal do Paraná, Curitiba 81531-980, PR, Brazil; 4 Department of Information Systems, Faculty of Social Sciences, University of Haifa, Haifa 31905, Israel

Computer vision and robotics are more and more involved in cultural heritage. From the data acquisition to heritage interpretation, the various tasks of the latter wide spectrum must face specificities of tangible and intangible heritages. For example, some ancient materials evolved with time and are possibly very unique, and thus are different from today’s buildings or paintings. Another example is the rareness of artefacts or cultural events as some dances or ceremonies practiced at a unique place on Earth by very few people, but it is still part of humanity’s heritage. The reality is even much wider than a few examples and inspires computer vision and robotics researchers to design new sensors, new robots, new methods and new interfaces in collaboration with historians, physicians, curators and teachers to allow archiving, analyzing and interpreting cultural heritage in an unprecedented way. The combination of so many various skills is now well known as digital heritage. This Special Issue has been organized to cover the recent research results about new imaging techniques, some of them involving robotics, to capture novel information of heritage artefacts, as well as data processing to detect and automatically analyze textual, pictural and building structures characteristics.

Imaging and data processing of cultural heritage artefacts is hard because of their rareness, sometimes due to their uniqueness. Hence, specific imaging sensors and technologies are developed to better document the heritage artefacts such as paintings. To this end, the article “Documenting Paintings with Gigapixel Photography” [1] of this Special Issue introduces a methodology to capture color images of ultra-high-definition and accurate chromaticity of historical paintings with applications on masterpieces of the Museo de Bellas Artes of Valencia, Spain, available online at https://gpix.webs.upv.es (accessed on 20 December 2022). Then, since paintings can exhibit different thicknesses on their surface or cracks for old ones, Reflectance Transformation Imaging (RTI) is used to highlight these non-flat characteristics. The RTI technique consists of capturing images from a single position by illuminating the artwork from various positions successively so that the shading helps to visualize even the tiniest variations of thickness. Contrary to existing techniques considering light domes or free-form manual lighting, the article “LightBot: A multi-light position robotic acquisition system for adaptive capturing of Cultural Heritage surfaces” [2] uses a robot arm to set the light source at various positions with accuracy so that the system is adaptive to the size of the artefact and other constraints. However, to capture characteristics of paintings that are beyond its surface, the article “Revealing hidden features in multilayered artworks by means of an epi-illumination photoacoustic imaging system” [3] proposes a photoacoustic imaging system enabling the capture of hidden layers of paintings.

Once data of heritage artefacts have been captured, their processing allows us to analyze ancient texts, such as those hand-written in ancient Greek tackled by the article “HTR (Handwritten Text Recognition) for Greek historical handwritten documents” [4] in which Recurring Neural Networks are trained to the transcription from new datasets prepared by its authors. The creation of datasets to train neural networks is a challenge with heritage artefacts since some objects to detect in images may suffer a rare representation with high variety of shape, such as medieval music instruments. This is the purpose of the article “Few-Shot Object Detection: Application to Medieval Musicological studies” [5] that is evaluated with several neural network architectures for object detection. On the other side of the object detection problem, the multiple object detection in 3D point cloud is the purpose of the article “Historic timber roof structure reconstruction through automated analysis of point clouds” [6], proposing an accurate solution to detect wooden structural elements that are highly repetitive in the same roof environment.

In the latter work, 3D point clouds are captured with Terrestrial Laser Scanner (TLS) but the so-called photogrammetry can output 3D points clouds from sets of images captured within the same environment, taking care of large overlap between them. The latter constraint inherent to enable photogrammetry is slightly relaxed by the advent of recent, low-cost, spherical cameras as those considered in the tutorial article “Use of low-cost spherical cameras for the digitization of Cultural Heritage structures into 3D point clouds” [7] of this Special Issue. The latter article is a nice illustration of the combination of emerging imaging technologies with adapted processing to ease the archiving work of heritage documenters, together with professional software available for concrete use on the ground by heritage interpreters.

Finally, this Special Issue offers also a snapshot of the international dimension of the digital heritage issue since the research institutions of the articles’ authors are in Spain, France, Switzerland, Greece, Italy and Austria. Of course, many other countries in the world are very active in digital heritage. Indeed, the editors of this Special Issue were also involved in organizing the “E-Heritage and Robotics” international workshop in conjunction with the IEEE/RSJ International Conference on Intelligent Robots and Systems (IROS) on October 2021 in Prague, The Czech Republic (see https://www.cvl.iis.u-tokyo.ac.jp/EHR2021, accessed on 20 December 2022) by committees of French, Japanese, Brazilian, Israeli and Italian researchers, among others, where the article about Lightbot [2] in this Special Issue was presented in addition to presentations of a panel of renowned researchers, archaeologists and a company owner from France, the USA and The Czech Republic. 
